# Complete Penetrance but Different Phenotypes in a Korean Family with Maternal Interstitial Duplication at 15q11.2-q13.1: A Case Report

**DOI:** 10.3390/children8040313

**Published:** 2021-04-20

**Authors:** Ji Yoon Han, Hyun Joo Lee, Young-Mock Lee, Joonhong Park

**Affiliations:** 1 Department of Pediatrics, College of Medicine, The Catholic University of Korea, Seoul 06591, Korea; han024@catholic.ac.kr; 2 Departments of Pediatrics, Yonsei University College of Medicine, Seoul 06273, Korea; genejoo@yuhs.ac; 3 Department of Laboratory Medicine, Jeonbuk National University Medical School and Hospital, Jeonju 54907, Korea; 4 Research Institute of Clinical Medicine of Jeonbuk National University-Biomedical Research Institute of Jeonbuk National University Hospital, Jeonju 54907, Korea

**Keywords:** maternal origin, interstitial duplication, 15q11.2-q13.1, schizophrenia, autism spectrum disorder, different phenotypes

## Abstract

The 15q duplication syndrome (dup15q) is due to the presence of at least one additional derived copy of the Prader–Willi syndrome/Angelman syndrome (PWS/AS) critical region that is approximately 5 Mb long within chromosome 15q11.2-q13.1. This report describes distinct roles of the origin of interstitial (int) dup15q underlining the critical importance of maternally active imprinted genes in the contribution to complete penetrance but different phenotypes of neuropsychotic disorders such as schizophrenia (SCZ) and autism spectrum disorder (ASD) in a Korean family. The proband’s mother as a consultant visited our hospital for her offspring’s genetic counseling and segregation analysis. She had two daughters diagnosed as SCZ or ASD and one son diagnosed as ASD. To resolve the potential genetic cause of SCZ and ASD in the proband and her sibling, whole genomic screening of chromosomal rearrangements by array-comparative genomic hybridization (CGH) was performed using SurePrint G3 Human CGH + SNP Microarray 4 × 180 K. Results of the array-CGH analysis revealed an interstitial duplication at 15q11.2-q13.1 (duplication size of 5.4 Mb) in the mother and her three offspring with SCZ or ASD. Our case, together with previous findings of high occurrence of psychotic disorder, suggest that maternally expressed gene product in the critical region of PWS/AS might mediate the risk of neurodevelopmental disorder (ASD) as well as psychotic disorder (SCZ). Multiple cytogenetic and molecular methods are recommended for investigating children with 15q11.2-q13.1 duplication and neuropsychotic disorders.

## 1. Introduction

The 15q duplication syndrome (dup15q) is due to the presence of at least one additional derived copy of the Prader–Willi syndrome/Angelman syndrome (PWS/AS) critical region that is about 5 Mb long within chromosome 15q11.2-q13.1 [1]. The occurrence of dup15q in the overall population is unclear. It might be as high as 1:5000 [2]. Particularly, in patients referred for medical genetic testing because of neurodevelopmental concerns (autism spectrum disorder, ASD; intellectual disability, ID; developmental delay, DD) or multiple congenital anomalies, the incidence of dup15q is about 1:508 [3]. The additional copy (or copies) most frequently arises by one of two principles: (1) an interstitial 15q11.2-q13.1 duplication (int dup15q) (typically one additional copy of 15q11.2-q13.1 within chromosome 15) that leads to trisomy for 15q11.2-q13.1; and (2) an isodicentric 15q11.2-q13.1 supernumerary chromosome (namely idic(15)) comprising typically two additional copies of 15q11.2-q13.1 that causes tetrasomy for 15q11.2-q13.1. Dup15q should be suspected in a patient with moderate to severe hypotonia in infancy and motor delays, ASD, DD/ID manifesting as speech and language delays, and infantile spasms. Dup15q resulting from maternal int dup15q has been found to be de novo in 85% and inherited from the mother in 15%. It is penetrant fully for ASD [4]. Rarely, dup15q may also be associated with psychotic disorder (such as schizophrenia, SCZ) and sudden unexplained death. Those with maternal idic(15) are affected more severely than those with an int dup15q [5].

Here, we describe a maternal origin of interstitial duplication at 15q11.2-q13.1 in the contribution to variable expressivity of neuropsychotic disorders such as SCZ and ASD in a Korean family

## 2. Case Presentation

**Figure 1:**
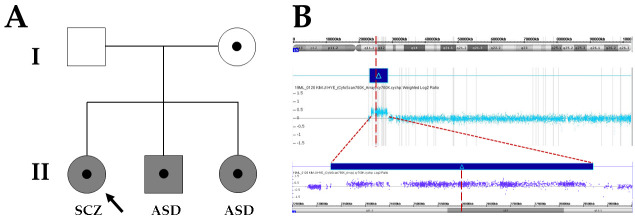
Pedigree analysis and array–comparative genomic hybridization in the proband. (**A**) Family pedigree depicting maternal origin of interstitial duplications at 15q11.2-q13.1 in a Korean family with schizophrenia and autism spectrum disorder. The grey symbol indicates affected individual. SCZ, schizophrenia; ASD: autism spectrum disorders. (**B**) Array–comparative genomic hybridization identifies interstitial duplications at 15q11.2-q13.1 between breakpoint 2 and 3 in the proband. The arr 15q11.2q13.1 (23,290,787_28,704,050) ×3 involving genes: *ATP10A*, *GABRA5*, *GABRB3*, *GABRG3*, *HERC2*, *MAGEL2*, *MKRN3*, *NDN*, *NPAP1*, *OCA2*, *PWRN1*, *PWRN2*, *SNHG14*, *SNORD115-1*, *SNORD116-1*, *SNRPN*, and *UBE3A*.

The proband’s mother (I-2 in Figure 1A), a 45-year-old female, visited the Department of Pediatric Neurology, Daejeon St. Mary’s Hospital (Daejeon, Korea) for her offspring’s genetic counseling and segregation analysis. Her development was completely normal through birth to adolescent without learning difficulties. She has four sisters and six nephews. None of them had a family history of neurodevelopmental delay, mental retardation, or psychotic disorders. The proband (II-1 in Figure 1A) was a 15-year-old female with neuropsychotic problems. She was referred for genetic diagnosis. She was the first child of non-consanguineous parents. The pregnancy was uneventful. Except for a history of ASD in her siblings, there was no history of neurological disorders including epilepsy, DD/ID, or psychotic disorders. She has neither facial dysmorphism nor anomaly. In childhood, her growth and developmental milestones were normal. After entering middle school, she began to show cognitive changes, immaturity, and worsen social communication that affected her ability to function in the school. She gradually developed problem behaviors with attention deficit and hyperactivity. She suffered from unpredictable agitation and bizarre posture. She seemed seeing or hearing somethings that did not exist. Effective communication was impaired and answers to questions were completely unrelated. Assessment for 15 years of age confirmed a diagnosis of SCZ. Since the diagnosis, she has been taking risperidone, clozapine, and fluoxetine. At age of 16, her intellectual quotient (IQ) was estimated to be 65, indicating mild ID. She is currently educated in a specialized school due to some problems in learning basic academic skills. The proband’s brother (II-2 in Figure 1A), a 13-year-old male, was the second child of non-consanguineous parents. The pregnancy was uneventful. When he first attended kindergarten, he was not interested in other children. He made unusual finger movements near his face and repeated some words without intent to communicate. He also had trouble starting a conversation. Assessment at 6 years of age confirmed a diagnosis of ASD. Since then, he has been taking risperidone. He attained an IQ of 60 on the Wechsler Intelligence Scale for Children Revised indicating mild intellectual disability. The proband’s sister (II-3 in Figure 1A), an 11-year-old female, was the third child of non-consanguineous parents. The pregnancy was uneventful. Early development was characterized by a delay in achieving fine motor, social-cognitive, and language milestones. At 5 years of age, she became aggressive and would hit her parents and throw objects. She displayed repetitive behaviors, including touching objects, opening and closing doors, and asking the same question many times. An assessment at 7 years of age confirmed a diagnosis of ASD. Since then, she has been taking methylphenidate and risperidone. Her IQ was estimated at 58.

Careful etiologic investigations of laboratory and radiology studies were performed. All results were within normal ranges for all of them (the proband and her siblings). Chromosomal analysis also revealed normal female karyotype. Fragile X testing was negative. Metabolic laboratory testing was within normal limits.

## 3. Results

Genomic DNA samples were obtained from leukocytes of peripheral blood using a QIAamp DNA Mini Kit (Qiagen GmbH, Hilden, Germany) according to the standard DNA isolation process. Their quantity and quality were estimated with a NanoDrop ND-1000 (ThermoFisher Scientific, Waltham, MA, USA) and a Qubit 1.0 (ThermoFisher Scientific), respectively. To resolve the potential genetic cause of SCZ and ASD in the proband and her sibling, whole genomic screening of chromosomal rearrangements by array-comparative genomic hybridization (CGH) was performed using SurePrint G3 Human CGH + SNP Microarray 4 × 180 K (Agilent Technologies, Santa Clara, CA, USA) according to the manufacturers’ instructions. All samples were matched with Human Genomic DNA reference (Promega, Madison, WI, USA). Data were obtained using Agilent Feature Extraction software 12.0.2.2 and Agilent CytoGenomics 4.0 and visually assessed using Agilent Genomic Workbench Software 7.0.4.0 and Agilent CytoGenomics 4.0. Copy number variations (CNVs) were identified using the ADM-2 algorithm with the following filters: minimal absolute average log ratio of 0.25 as a cut-off, >5 Mb of copy number neutral loss of heterozygosity regions, and minimal size of 200 Kb in region. Genomic positions were mapped using the human genomic reference sequence GRCh37/hg19. As a result, array-CGH analysis revealed an interstitial duplication at 15q11.2-q13.1 (duplication size of 5.4 Mb), in the proband and her siblings. Because the diagnosis of dup15q is established by identification of at least one extra maternally derived copy of the PWS/AS critical region, genetic counseling and segregation analysis were recommended for the proband’s parents. Same interstitial 15q duplication was detected only in her mother (Figure 1B). Other rare CNVs classified as likely pathogenic were not identified in the proband and her family members.

Since clinical manifestation of the proband differs from her siblings, additional molecular analysis at the nucleotide level was performed in the proband only. Exomic DNA of the proband was enriched using the Agilent’s SureSelect XT Human All Exon v5 (Agilent Technologies) and paired-end sequenced using an Illumina HiSeq2500 (Illumina, San Diego, CA, USA) for detection of the variant considering possible familial disease. Base calling, alignment, variant calling, annotation, and quality control reporting were performed using a GATK Best Practices workflow for germline short variant discovery and manually reviewed by medical laboratory geneticists. However, whole exome sequencing (WES) identified no likely pathogenic variants that may correlate with clinical phenotypes such as ID or SCZ of the proband.

## 4. Discussion

The dup15q may be one of the most common single genetic causes of ASD besides Fragile X syndrome [6]. Although penetrance appears to be complete in maternal int dup15q, some patients may show mild clinical manifestations and appear to be unaffected, reflecting different phenotypes rather than true non-penetrance. However, penetrance of maternal idic(15) always occurs with variable expressivity [7]. PWS/AS critical region is always included in the idic(15) or the interstitial duplications which lead to dup15q. When the PWS/AS critical region is imprinted, maternally derived increases in copy number lead to dup15q, whereas paternally derived increases are usually related to more diverse and variable neurodevelopmental symptoms [8]. The degree of clinical symptom varies even among patients with the same genetic mechanism due to a gene dosage effect, by deletion or duplication. Some phenotypic characteristics such as ASD are observed more consistently in patients with a maternal large int dup15q that is more than 5 Mb long or idic(15) which extends beyond the PWS/AS critical region [8,9].

On the other hand, Phenotypes resulting from paternal and maternal duplications can differ, because this duplicated region is imprinted. In our siblings, the maternal int dup15q resulted in different phenotypes of neuropsychotic disorders such as SCZ and ASD in a Korean family. Interestingly, the mother is normal. However, her three offspring showed SCZ or ASD, although all of them carried int dup15q. The relative risk to the proband’s sibling depended on the genetic status of the proband’s mother. If the mother carrying int dup15q inherited it from her father, she might appear to be unaffected or show characteristics related to paternal duplications. These characteristics might share some similarities, although they are distinct from those of the proband. However, it is not possible to reliably predict the degree of phenotypic severity in the patient with dup15q [7]. Unfortunately, the origin of the mother’s int dup15q in our case was not determined because array-CGH was not available for her parents.

Typical clinical manifestations of dup15q usually include ASD, language impairments, DD/ID, and seizures [10]. Problematic behaviors including shouting, tantrums, aggressiveness, repetitive movements, and stereotypies are often found. Particularly autistic characteristics might be present in up to 50% of affected patients [8]. Characteristics of ASD, particularly difficulties with social interaction, might be frequent between early and late childhood [11]. Conversely, psychotic disorder associated with a mood disorder with a high occurrence of ASD in dup15q might be misdiagnosed as SCZ. Psychotic disorder is not a commonly ascertained comorbidity in dup15q [12]. However, it is a common comorbidity in PWS resulting from uniparental disomy that involves the maternal origin of dup15q similarly [13]. There are somewhat overlapping similarities in clinical features among 15q11-q13 duplication, PWS, and AS (Table 1) [14,15,16,17,18,19,20,21]. However, SCZ is not a common clinical manifestation associated with the 15q11-q13 region. Pediatric patients with early onset of SCZ should be considered for duplication of this region. These patients tend to have higher verbal and cognitive abilities than those with dup15q. Our case, together with previous findings of high occurrence of psychotic disorder [22], suggest that maternally expressed gene products in the critical region of PWS/AS might mediate the risk of neurodevelopmental disorder (such as ASD) and psychotic disorder (such as SCZ). Because there are some overlaps in phenotypic characteristics of maternal and paternal duplications, parent of origin testing should be estimated to determine whether the proband has a maternal dup15q syndrome or a paternal interstitial duplication [8].

**Figure 2:**
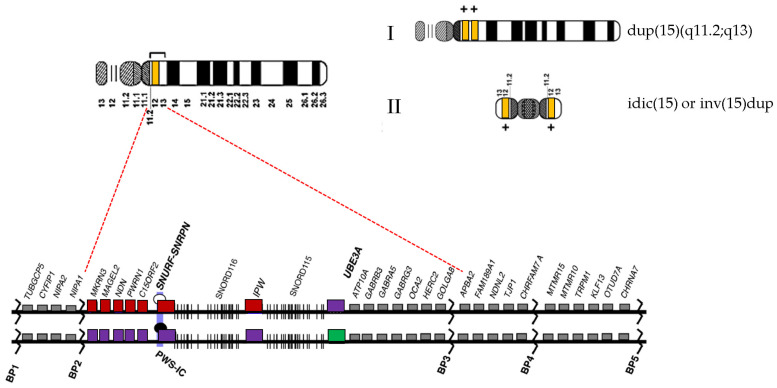
Map of 15q11.2-q13 region. (**I**) Interstitial duplication 15q11.2-q13 and (**II**) idic(15) or inv(15)dup. Gene distribution between breakpoint (BP) 1 and BP5 on the proximal 15q region. 15q11.2-q13.1 region is located between BP2 and BP3. Genes displayed by red and green boxes are imprinted and expressed from paternal and maternal alleles, respectively. Violet box indicates silenced allele. Gray box marks genes expressed from both parental alleles. BP, break point; PWS, Prader–Willi syndrome; IC, imprinting center.

On the other hand, WES is a comprehensive approach for identifying novel candidate genes and for suggesting de novo mutations that might contribute substantially to the genetic risk for ASD [23] and SCZ [24]. Genomic instability of proximal 15q including five regions of low copy repeats or segmental duplications designated by breakpoints (BPs) can lead to higher susceptibility to genomic rearrangements [4]. Several genes crucial for brain development and synaptogenesis, such as small nuclear ribonucleoprotein polypeptide N (*SNRPN*), ubiquitin protein ligase E3A (*UBE3A*), and three gamma-aminobutyric acid (GABA) type-A receptor genes (*GABRA5*, *GABRB3*, and *GABRG3*), have been reported [25]. A part of the PWS/AS critical region between BP2 and BP3 involves several paternally expressed genes (including *MKRN3*, *MAGEL2*, *NDN*, *PWRN1*, *C15ORF2*, and *SNRPN*) and two maternally expressed genes (*UBE3A* and *ATP10A*) (Figure 2) [26].

## 5. Conclusions

In conclusion, this report describes distinct roles of origin of int dup15q, underlining the critical importance of maternally active imprinted genes in the contribution to complete penetrance but different phenotypes of neuropsychotic disorders such as SCZ and ASD in a Korean family. Multiple cytogenetic and molecular methods are recommended for investigating children with 15q11.2-q13.1 duplication and neuropsychotic disorders.

## Figures and Tables

**Table 1:** Comparison of clinical features of Prader–Willi, Angelman, and 15q11.2-q13.1 duplication syndromes and our patients [14,15,16,17,18,19,20,21].

Clinical Features	PWS	AS	Dup15q	II-1	II-2	III-3
Prevalence	1/15,000–30,000	1/12,000–20,000	Unknown			
Hypotonia	+++	+	++	Absent	Absent	Absent
Developmental delay	++	+++	++	Absent	Present	Present
Intellectual disability	+	+++	++	Present	Present	Present
Autism spectrum disorders	+	-	+	Absent	Present	Present
Behavioral problems	++	+	++	Present	Present	Present
Tremor and/or ataxia	-	+++	-	Absent	Absent	Absent
Sleep disturbance	++	+	-	Absent	Absent	Absent
Seizures	-	++	+	Absent	Absent	Absent
Microcephaly	-	++	-	Absent	Absent	Absent
Strabismus	-	+	-	Absent	Absent	Absent
Failure to thrive	+++	-	-	Absent	Absent	Absent
Feeding difficulty	+++	-	-	Absent	Absent	Absent
Hypogonadism	+++	-	-	Absent	Absent	Absent
Hyperphagia	+++	-	-	Absent	Absent	Absent
Obesity	+++	-	-	Absent	Absent	Absent
Short stature/reduced growth	++	-	+	Absent	Absent	Absent
Characteristic facial features	++	-	+	Absent	Absent	Absent
Small hand/feet	++	-	-	Absent	Absent	Absent
Happy demeanor	-	+++	-	Absent	Absent	Absent
Hyperpigmentation	-	-	+	Absent	Absent	Absent
Hypopigmentation	++	-	-	Absent	Absent	Absent

PWS, Prader–Willi syndrome; AS, Angelman syndrome; Dup15q, 15q11.2-q13.1 duplication syndrome; +++, consistent (100%); ++, frequent (80%); +, related (20–80%).

## Data Availability

Data presented in this study are available from the corresponding author upon reasonable request.
